# Community and partner engagement in dissemination and implementation research at the National Institutes of Health: an analysis of recently funded studies and opportunities to advance the field

**DOI:** 10.1186/s43058-023-00462-y

**Published:** 2023-07-12

**Authors:** Aubrey Villalobos, Dara Blachman-Demner, Antoinette Percy-Laurry, Deshiree Belis, Manami Bhattacharya

**Affiliations:** 1 Division of Cancer Control and Population Sciences, National Cancer Institute, 9609 Medical Center Dr, Bethesda, MD 20892 USA; 2 Office of Behavioral and Social Sciences Research, Office of the Director, National Institutes of Health, 31 Center Dr, Bethesda, MD 20892 USA; 3 Office of Science Policy, Planning, Evaluation and Reporting, National Institute on Minority Health and Health Disparities, 6707 Democracy Blvd, Bethesda, MD 20817 USA

**Keywords:** Community engagement, Partner engagement, Health disparities, Health equity, Dissemination and implementation

## Abstract

**Background:**

As the focus has grown in recent years on both engaged research and dissemination and implementation (D&I) research, so too has federal funding to support these areas. The purpose of this analysis is to provide an overall perspective about the range of practices and approaches being used to engage partners in D&I research, with special attention to disparities-relevant research, and to identify gaps and opportunities in research funded by the US National Institutes of Health (NIH) in this space.

**Methods:**

This analysis examined a portfolio of active D&I research grants funded in fiscal years 2020 and 2021 across the NIH. Grant applications were deductively coded and summary statistics were calculated. Cross-tabulations were used to identify trends by engagement and disparities foci.

**Results:**

There were 103 grants included in the portfolio, of which 87% contained some form of community or partner engagement, and 50% of engaged grants were relevant to health disparities. Engagement was planned across the research continuum with each study engaging on average 2.5 different partner types. Consultation was the most common level of engagement (56%) while partnership was the least common (3%). On average, each study used 2.2 engagement strategies. Only 16% of grants indicated formally measuring engagement. Compared to non-disparities studies, disparities-relevant studies were about twice as likely to engage partners at the higher levels of partnership or collaboration (19% vs. 11%) and were also more likely to be conducted in community settings (26% vs. 5%).

**Conclusions:**

Based on this portfolio analysis, D&I research appears to regularly integrate engagement approaches and strategies, though opportunities to deepen engagement and diversify who is engaged remain. This manuscript outlines several gaps in the portfolio and describes opportunities for increasing engagement to improve the quality of D&I research and application to advancing health equity. In addition, opportunities for leveraging the consistent and systematic application of engagement approaches and strategies to advance the science of engagement are discussed.

Contributions to the literature

- This analysis of D&I research grants funded by the National Institutes of Health (NIH) provides evidence that community and partner engagement is common.
- These findings contribute to a gap in the literature by describing the extent and qualities of engagement activities in NIH-funded D&I research and the intersection of engagement and disparities-relevant research in the D&I portfolio.
- These findings also contribute to a gap in the literature by outlining opportunities to increase and deepen engagement efforts in support of NIH’s commitment to health equity and NIH’s mission to generate knowledge and the application of that knowledge to enhance health.

## Background

In recent years, it has become increasingly clear that for efficacious health care and public health interventions to be effectively implemented, broadly scaled up, and sustained over time, there needs to be increased appreciation for and investment in areas of scientific inquiry that can provide insight and perspectives as to how to best reach these goals. Specifically, leveraging the behavioral and social sciences, community and partner engagement, health equity, and implementation science is critical to advance such efforts.

Dissemination and Implementation (D&I) research at the US National Institutes of Health (NIH), under the umbrella of implementation science, has evolved over the past few decades. As one of the largest funders of D&I research, NIH now has a standing set of funding opportunities [1–3], a standing study section [4], an annual conference [5], dedicated D&I-focused staff, and ongoing training programs for extramural investigators (e.g., Training in D&I Research in Cancer [TIDIRC]) [6]. In addition, most NIH Institutes, Centers, and Offices feature some aspect of D&I research in strategic plans or initiatives as there is an increasing recognition of the value of implementation science approaches to facilitating effective uptake, sustainability, and scale up of efficacious medical and behavioral interventions to improve population health.

Several recent efforts have recognized the importance of integrating the methods and approaches of community engagement with D&I research to enhance rigor and relevance and to address health disparities. A special issue of *Translational Behavioral Medicine* highlighted research gaps and opportunities and included a reflection and historical perspective on NIH efforts in this space [7, 8]. There appears to be broad interest in bringing not just community, but also other partner engagement into D&I research. This is evidenced by an increasing number of perspective pieces and commentaries highlighting the potential benefits of this integration to both research and practice, outlining examples of federal efforts to support this research, and offering of potential frameworks to accomplish that integration [9–13]. There have also been a few published case studies documenting interventions that worked to integrate engagement in D&I research for a diverse range of health and related outcomes including violence, heart disease, and cancer [14–16]. A recent scoping review by Triplett and colleagues identified 103 child mental health evidence-based treatment implementation projects that included community or partner engagement [17]. Triplett et al. [17], and others [18], have suggested that integration of engagement methods and approaches into D&I research could lead to improved implementation and health outcomes, but this has yet to be documented in many cases.

One of the major lessons learned from the experiences during the COVID-19 pandemic is that even with the best scientific advancements (such as vaccines), these life-saving interventions will not be widely taken up and scaled without a concerted effort to understand and deploy implementation strategies that match each unique setting, context, and population. A primary goal of D&I research is to achieve broad, sustainable use of an evidence-based program, policy, or practice, which requires inclusivity and shared ownership and buy-in from a range of partners and community members. As a result of this understanding, the NIH launched two major COVID efforts designed to focus on community engagement, partnerships, and D&I approaches in order to engage populations most at risk and who simultaneously face the most barriers to access and participation in COVID testing (Rapid Acceleration of Diagnostics-Underserved Populations) [19] and vaccine uptake (Community Engagement Alliance against COVID-19) [20]. Further, in 2022, a new NIH Common Fund initiative was launched, Community Partnerships to Advance Science for Society (ComPASS), which focuses on advancing health equity research through community-driven, multisectoral structural interventions to reduce health disparities. Though not an implementation science initiative per se, the focus on sustainability and structural level change over time means that a number of implementation models, processes, and outcomes are likely to be incorporated [21].

The examples above notwithstanding, there remain clear and notable gaps in community and partner engagement in D&I research. There do not appear to be many “standard” D&I studies that routinely and rigorously integrate community or partner engagement approaches into their work, and engagement is not well-integrated into most D&I theories, models, or frameworks [22]. Further, implementation scientists may not have the competencies necessary to lead engaged or participatory research [23]. Given increased recent public attention to health disparities following the highly publicized racial/ethnic COVID-19 disparities, it seems timely and imperative that D&I research be done in partnership with the communities that are most in need and the organizations that serve them. Yet, D&I research often fails in this regard. Indeed, a recent reflection on “promises and pitfalls in implementation science” identifies this lack of connection with community partners and lack of focus on health equity as fundamental to the field’s challenges [24]. These identified pitfalls include re-creation of the research to practice gap, misalignment of timelines, incentives, and priorities with community partners, and complexity of strategies that may not match community needs. These authors offer several ways forward, many of which involve increased attention to better integration of engagement approaches to inform D&I efforts since community engaged research is rooted in social justice and advancing health equity [25–27]. Similarly, in their scoping review, Triplett et al. found that the engagement across 103 projects was often shallow and lacking shared power in decision-making [17]. They called for increased effort to improve engagement to proactively address barriers and increase likelihood of successful implementation.

Given the increased attention to health disparities and health equity in recent years, particularly since the start of the COVID-19 pandemic in 2020, and calls to increase engagement in D&I research, several initiatives across multiple NIH Institutes, Centers, and Offices over the last several years have attempted to stimulate the field. These initiatives invest in programs of research that seek to bring community and partner engagement together with D&I research to tackle entrenched health disparities and challenges across topics such as cancer [28–30], HIV [31, 32], opioid addiction [33, 34], and environmental health [35]. In addition, the Clinical and Translational Science Award (CTSA) Program has long prioritized engaging communities in the research process, but has recently increased their emphasis on diversifying who is being engaged and expanded their integration of D&I research into their infrastructure and function [36, 37]. These efforts are promising, yet we currently lack a comprehensive understanding of how NIH investments are working to support this integration, whether they are in fact successfully stimulating the field to conduct studies that increasingly incorporate and integrate engaged research approaches in D&I research more broadly. Such an understanding would allow us to identify gaps and opportunities to further stimulate and support the field and ultimately to produce more rigorous, relevant, and impactful science.

The purpose of this analysis is to provide insight into the current range of practices and approaches being used to engage communities and partners in NIH-sponsored D&I research, with special attention to disparities-relevant research, and to identify gaps and opportunities. This analysis focuses on new research funded in fiscal years 2020 and 2021, the most current complete data available at the time of analysis, to evaluate whether recent efforts to promote community engagement in research across NIH through specific initiatives and conversations such as those mentioned above have extended into the general NIH-funded D&I portfolio, which given its size may be a proxy for the D&I field more broadly. To that end, the research questions we sought to answer were as follows:

1. To what extent and how are community members and other partners engaged in recent NIH-funded D&I research?
2. Are there common themes that emerge with respect to engagement across implementation phases, strategies, or other key domains?
  a To what extent are NIH-funded engaged D&I research studies relevant to health disparities?
  b How do NIH-funded D&I research studies relevant to health disparities involve community or partner engagement?

## Methods

This study followed the Strengthening the Reporting of Observational Studies in Epidemiology (STROBE) guideline for reporting observational studies since it was examining a cross-section of grants that met specified eligibility criteria [38, 39]. This analysis included active research grants funded in fiscal years 2020 and 2021 across all NIH Institutes, Centers, and Offices that were funded through the dedicated D&I funding opportunities [1–3] or reviewed in the Science of Implementation in Health and Healthcare (SIHH) [4] (Dissemination and Implementation Research in Health (DIRH) through 10/2020) study section. An internal NIH portfolio analysis platform, *iSearch,* was used, which provides comprehensive, easy-to-use access to carefully curated, extensively linked datasets of funded and unfunded grant applications, historic and current.

The study team developed a codebook directly stemming from the research questions through an iterative process that included review of other NIH-specific portfolio analysis codebooks and publications, a review of the literature on D&I and community and partner engagement, and discussions among the study team and collaborators. Table 1 shows data extracted from grants. Grant characteristics were extracted from the metadata in *iSearch.* Variables coded captured information about the condition under study and details regarding the intervention under study including the social ecological level(s) targeted [40, 41] and where and by whom they are delivered. Codes also recorded details about the study design and methods [42–44], implementation phase, implementation strategies used [45, 46], and use of theories, models, and frameworks [47, 48]. Grants were coded for where on the research continuum (e.g., intervention design, evaluation) and through what approaches (e.g., practice-based research [49], community-based participatory research (CBPR) [50]) engagement was undertaken. Grants were also coded for the planned audience for engagement according to the 7Ps framework proposed by Concannon et al. [51] with definitions adapted from the Patient Centered Outcomes Research Institute (PCORI) [52], the highest level of engagement described according to the continuum proposed by Sanders Thompson et al. [53], and any indicators of equity in engagement the proposal described, drawing on concepts discussed by Key et al. [54] (see Table 1 for definitions). Grants were also coded as disparities-relevant if they related to minority health or health disparities, as defined by the National Institute on Minority Health and Health Disparities [55]. The codebook was programmed into *iSearch*’s curation tool and piloted on two grants by two members of the study team.

**Table 1:** Data extraction

**Information extracted**	**Description**
Funding ICO^a^	NIH Institute, Center, or Institute [ICO] funding the grant
Study Section^a^	Scientific review group that scored the grant application
RFA PAR #^a^	Notice of funding to which the grant was submitted
Funding Mechanism^a^	Type of grant
Fiscal Year^a^	Fiscal year in which the grant was funded
Intervention	Is an intervention being disseminated, implemented or de-implemented in this study? Take implementation to also mean de-implementation throughout coding. Take intervention to mean evidence-based program, practice, policy. (yes or no)
Intervention Level	Social ecological level at which the study intervention takes place [40, 41]
Intervention Setting	Setting in which the intervention is delivered
Intervention Implementer(s)	Roles implementing the intervention
Condition Under Study	Conditions are diseases, disorders, syndromes, illnesses, or injuries that are automatically extracted from grant text using natural language processing software that identifies phrases and synonyms along with their associated MeSH semantic type.
Health Disparities-Relevant Research	Are minority health, health disparities, or health equity being studied? (yes or no) **• Minority health research** is the scientific investigation of distinctive health characteristics and attributes of minority racial and/or ethnic groups who are usually underrepresented in biomedical research to understand health outcomes in these populations [55]. **•** A health disparity is a health difference that adversely affects disadvantaged populations, based on higher disease burden, risk factors, condition-specific symptoms, and/or other categories of health outcomes. **Health disparities research** is directed to understanding the mechanism as to why a defined disadvantaged group has a worse health outcome compared to a reference group and how this knowledge is translated into interventions to reduce health disparities [55]. **•** Health equity means all populations will have an equal opportunity to live long, healthy, and productive lives. **Health equity research** is directed to upstream, fundamental causes of health disparities at outer social ecological levels (community, environment, policy), including interventions to address social or structural determinants of health.
Disparities Population	Population with health disparities that is the focus of the research. For NIH, populations that experience health disparities include [55]: **•** Racial and ethnic minority groups (see OMB Directive 15) **•** People with lower socioeconomic status (SES) **•** Underserved rural communities **•** Sexual and gender minority (SGM) groups
Engagement Timing	Where is engagement happening throughout the study?
Engagement Audience	Types of research participants and partners engaged, using the The 7Ps Framework from Concannon et al. (2012) and definitions adapted from PCORI (2018) [51, 52] **• Direct service providers or practitioners**—professionals who would deliver the intervention/service and/or the organizations they work within **• Patients / survivors or their families**—people who would receive the intervention/service, e.g., persons with current or past experience of illness or injury, family members or other unpaid caregivers of patients **• Policymakers**—those who help craft public policy at any level of government, including federal, state, and local government officials; federal, state, and local units of government; and organizations that represent policymakers **• Product makers / intervention designers**—people or companies who design, invest in, or manufacture the original innovation/intervention being implemented, e.g., drug, diagnostic technology, device, electronic records or other software or app developers or manufacturers and organizations representing the life sciences industry **• Program/system administrators**—people or organizations who would approve the adoption of the intervention or service **• Public / General community members**—people who have not received a disease diagnosis who would potentially receive the intervention/service, e.g., school children or their parents, people with lived experience in determinants of health outcomes, consumer advocacy organizations **• Purchasers or Payers**—people or organizations who would pay for the intervention or service through underwriting or reimbursement, serving as financial intermediaries, or purchasing health benefits for employees and their dependents, e.g., could include a foundation sponsor/funding agency; individual businesses as well as local, state, regional, and national business groups, coalitions that represent businesses, and health coalitions; private insurers and public insurers, and organizations representing insurers **• Partner organizations**—organizations that can provide access to any of the above groups, e.g., coalitions/networks, professional associations, advocacy groups, etc.
Engagement Level	Highest level of engagement demonstrated in proposal according to definitions from Sanders Thompson et al. [53]: **• Outreach and education**—Research team members develop, implement, and evaluate strategies to reach the population of interest. Organizational partners can be engaged as advisors and can make key connections. In some instances, researchers are trying to educate community residents and/or patients about a particular topic. In these cases, outreach efforts are used to gain audiences for education sessions and/or materials. **• Consultation**—Researchers ask community residents and/or patients for advice on important elements of a project or activity. The provided feedback informs the research, but the researchers are responsible for designing and implementing projects with no help expected from the people who were consulted. **• Cooperation**—Researchers ask community residents and/or patients for advice and help with a project. Such help may include activity in defined aspects of the project, including recruitment, activities related to doing the intervention, the creation of study questions and measures, and the interpretation of outcomes. Researchers and community residents and/or patients work together to make decisions throughout the project. **• Collaboration**—Patients, caregivers, clinicians, researchers, and/or community members partner in every aspect of the research, including setting priorities, study design, implementation, analysis/interpretation, and dissemination. Collaborations are built on mutual respect and trust. All partners are valued, benefit from the research, and share decision-making, power, and resources. **• Partnership**—A strong, bidirectional relationship exists among patients, caregivers, clinicians, researchers, and community members (or a combination of these categories) regarding every aspect of the research, including setting priorities, study design, implementation, analysis/interpretation, and dissemination. The relationship is built on trust and mutual respect. All partners are valued, benefit from the research, and share decision-making, power, and resources. Strong partnership processes exist for how resources are shared, how decisions are made, and how ownership of the work is determined and maintained. Partnerships are the result of long-term relationships and have moved beyond working on a single project. Partners have a history of collaboration, having worked together previously.
Engagement Approach	Engagement approach described
Engagement Strategies	Engagement methods/ strategies/ activities described
Engagement Equity	Indicators of equity in engagement based on Key et al. [54], definitions original: **• Decision-making**—Proposal describes shared decision-making authority and/or processes to facilitate equitable, engaged consensus building for decision-making and conflict resolution **• Influence**—Proposal describes processes or mechanisms for how practitioner(s) can inform the research at any stage (e.g., from question and hypothesis generation to study design and implementation to data analysis and knowledge creation to dissemination and translation of results) **• Mutual benefit**—Proposal describes how the engagement and/or study outcomes will be of value to both the researcher(s) and community partner(s) **• Ownership**—Proposal addresses who has ultimate ownership over the research, data collected and/or the distribution of findings **• Power and control**—Proposal recognizes power differentials between academic and community partners and describes actions to mitigate, including but not limited to detailing specific leadership roles for partner(s) **• Resource-sharing**—Evidence of financial resource allocation (e.g., subaward(s)) with community partner organization(s) in budget commensurate with role in the project (e.g., community co-investigator or multi-PI) **• Responsibility**—Proposal describes specific responsibilities of research team to the community partner(s) (e.g., capacity building)
D&I Study Type	Is this a dissemination, implementation, both D&I, or de-implementation study? **• Dissemination research** is the scientific study of targeted distribution of information and intervention materials to a specific public health or clinical practice audience. The intent is to understand how best to spread and sustain knowledge and the associated evidence-based intervention(s) [1–3]. **• Implementation research** is the scientific study of the use of strategies to adopt and integrate evidence-based health interventions into clinical and community settings in order to improve patient outcomes and benefit population health [1–3]. **• De-implementation** is “reducing or ceasing the delivery of ineffective, unproven, harmful, or low-value practices, treatments, programs, interventions, and guidelines” [56]
Implementation Phase	Phase(s) of implementation the research questions address
Study Design	Study design employed
Hybrid Study Type	From Curran et al. [44]
D&I Theory	Model, theory or framework used
Implementation Strategies	Implementation strategies employed, as defined in Waltz et al. and Powell et al. [45, 46]

^a^Automatically extracted as part of grant metadata

Full-text grant proposals were accessible through *iSearch.* Each grant proposal (research summary, human subjects, and budget justifications sections) was independently coded directly in *iSearch* by two assigned coders. Coders (*N* = 21) were generally assigned to grants from their own Institutes, with additional support provided by the NIH Office of the Director’s Division of Program Coordination, Planning, and Strategic Initiatives. Training for coders on how to use the *iSearch* platform was provided by the NIH Office of Portfolio Analysis. A subsequent training was held by the study team via videoconference to orient coders to the study purpose and review codebook variables and definitions. The recording of the training was made available to all coders to refer to throughout the coding period. The study team held optional “office hours” via video conference for coders three times over the course of the coding period to answer questions and offer clarifications about code definitions to facilitate reliability. The office hours recordings were distributed to all coders via emails that also included notes on important coding clarifications and examples reviewed during the office hour discussions. Coding discrepancies were resolved through discussion to consensus between the coding pairs and amended in *iSearch*. If intercoder consensus could not be reached, a third coder from the study team reviewed the grants and assisted in resolving disagreements, though this occurrence was rare given the clarifications provided via office hours and emails. Summary analyses, including descriptive statistics, were used to answer the first research question and bivariate cross-tabulations were used to answer the second research question. All analyses were conducted in a Microsoft Excel database exported from *iSearch*.

## Results

A total of 103 grants met the inclusion criteria and were funded by 16 different NIH Institutes, Centers, and Offices (Table 2). A majority (57%) of grants were funded through the DIRH funding opportunities [1–3], 87% were reviewed by the SIHH/DIRH study section, and most were R01s (64%) or R21s (22%). Ten grants did not involve interventions and rather were solely focused on developing databases, conducting modeling, developing or validating measures, retrospectively studying policies or grants portfolios, or were in early pre-implementation exploratory phases prior to developing an intervention. Over two-thirds of grants (69%) targeted two or more levels of the social-ecological model; predominantly the individual (66%) and organizational (62%) levels, with the fewest targeting the societal level (6%). The most common intervention settings included healthcare organizations (67%) and the community (18%). Accordingly, common intervention implementers included healthcare providers (63%), lay health personnel (17%), clinic support staff (15%), and clinic administrators (13%). Less common implementers included policymakers (4%), social service workers (5%), public health professionals (7%), and implementation support practitioners like knowledge brokers, technical assistance providers, or practice facilitators (8%).

**Table 2:** Characteristics of included grants (*N* = 103)

	Number (%)
Funding ICO	
NCI	27 (26)
National Heart, Lung, and Blood Institute	17 (17)
National Institute of Mental Health	16 (15)
National Institute on Drug Abuse	11 (11)
Eunice Kennedy Shriver National Institute of Child Health and Human Development	9 (9)
Others	23 (22)
Study section	
DIRH/SIHH	90 (87)
Others	13 (13)
RFA PAR #	
DIRH funding opportunities	59 (57)
Others	44 (43)
Funding mechanism	
R01	66 (64)
R21	23 (22)
R37	6 (6)
Others	8 (8)
Fiscal year	
2020	48 (47)
2021	55 (53)
Intervention, yes	93 (90%)
Intervention level^a^	
Individual	68 (66)
Interpersonal	24 (23)
Organizational	64 (62)
Community	19 (18)
Societal	6 (6)
Intervention setting^a^	
Community setting or community-based organization	19 (18)
Faith-based organization	4 (4)
Health department	2 (2)
Healthcare	69 (67)
Home	8 (8)
Justice/legal	2 (2)
Online or mobile phone	5 (5)
School	6 (6)
Workplace	1 (1)
Other	3 (3)
Intervention implementers^a^	
Clinic administrator/manager	13 (13)
Clinic support staff	15 (15)
Educator or school health personnel	11 (11)
Healthcare provider	65 (63)
Implementation support practitioner/professional	8 (8)
Lay health personnel	18 (17)
Policymaker	4 (4)
Public health practitioner/professional	7 (7)
Social service worker	5 (5)
Others	23 (22)
Health disparities-relevant research, yes	47 (46)
Disparities population^a^	Number (% out of* n* = 47)
Racial or ethnic minorities	22 (47)
Underserved rural	18 (38)
Socioeconomically disadvantaged	22 (47)
Sexual or gender minorities	6 (13)
Other	4 (9)

^a^Responses not mutually exclusive

Table 3 shows that most studies (87%, *n* = 90) included some form of community or partner engagement. Engagement was planned across the research continuum, with formative research (61%) and implementation strategy design (52%) being the most common and theory development (6%) being least common. On average, each study engaged 2.5 different partner types with direct service providers (77%) and patients/survivors (54%) being the most common, and product makers (3%) and purchasers or payers (7%) being the least common. The highest level of engagement ranged Sanders Thompson et al.’s [53] continuum with consultation being the most common (56%) and partnership (3%) and outreach (4%) being the least common. Practice-based research was a common approach (23%), as was CBPR (16%), while one-third of studies did not specify any particular approach to engagement. On average, each study used 2.2 engagement strategies, with the most common being key informant data collection (77%) and project advisory groups (39%). Nearly half (48%) of studies did not describe any indicators for equitable engagement, but for those that did, influence (28%) and mutual benefit (26%) were most clearly articulated. Finally, only 16% of engaged research grants reported formally measuring engagement.

**Table 3:** Characteristics of the engagement in included grants

When is engagement planned?^a^	Number (% out of *N* = 103)
In theory development	6 (6)
In intervention design	32 (31)
In implementation strategy design	54 (52)
In formative research	63 (61)
In evaluation and measurement	34 (33)
Only stated but not explained in depth	5 (5)
Other	6 (6)
Not at all	13 (13)
Who will be engaged?^a^	Number (% out of n = 90)
Direct service providers or practitioners	69 (77)
Partner organizations	29 (32)
Patient/survivors or their families	49 (54)
Policymakers	16 (18)
Product makers/intervention designers	3 (3)
Program/system administrators	33 (37)
Public/General community members	19 (21)
Purchasers or payers	6 (7)
Other	6 (7)
Highest level of engagement in study	
Outreach and education	4 (4)
Consultation	50 (56)
Cooperation	21 (23)
Collaboration	12 (13)
Partnership	3 (3)
Engagement approach	
Practice-based research	22 (23)
Community-based participatory research (CBPR)	14 (16)
Community engaged research (CEnR)	10 (11)
Other	13 (14)
Not specified	31 (33)
Engagement strategies^a^	
Brainstorming/crowdsourcing ideas	4 (4)
Capacity-building	20 (22)
Human-centered design	10 (11)
Information dissemination	15 (17)
Key Informant data collection in needs assessment, formative research, or research agenda setting	69 (77)
Group meetings for consensus building or prioritization	20 (22)
Process, system, intervention, or implementation mapping	8 (9)
Project steering committee, advisory group or other decision-making body	35 (39)
Partners as active members of research team	15 (17)
Others	3 (3)
Indicators of equity in engagement^a^	
Decision-making	14 (16)
Influence	25 (28)
Mutual benefit	23 (26)
Ownership	0 (0)
Power and control	1 (1)
Resource-sharing	13 (14)
Responsibility	2 (2)
Not described	43 (48)
Is engagement measured? yes	14 (16)

^a^Responses not mutually exclusive

In looking at themes that emerged with respect to engagement and key implementation domains, studies that were categorized as D&I or de-implementation all involved engagement at some level, whereas 14% (*n* = 11) of the grants categorized as implementation-only studies had no engagement (Table 4). Similarly, 12% (*n* = 11) of studies coded as involving an intervention reported no engagement (data not shown in table). Compared to grants without community or partner engagement, engaged research projects were more likely to be focused on implementation phases of testing strategies (61% vs. 31%), scaling up (20% vs. 8%), and/or sustainability (19% vs. 8%). Grants involving engagement were more likely to use a case study (3% vs. 0%) or observational design (37% vs. 23%) and non-engaged grants were more likely to use modeling (15% vs. 7%); there was no substantial difference in the use of experimental or quasi-experimental designs. All grants set in the community or within a community-based organization involved some level of engagement (data not shown in table). Grants involving community or partner engagement appeared more likely to leverage less-common D&I theories, models, and frameworks or those from other disciplines compared to grants without engagement (60% vs. 23%). Compared to grants without community or partner engagement, engaged research projects were more likely to apply the following implementation strategies: adapt and tailor to context (50% vs. 38%), develop interrelationships (36% vs. 23%), engage consumers (40% vs. 8%), provide interactive assistance (32% vs. 23%), support clinicians/service providers (29% vs. 8%), train and educate (48% vs. 23%), and use evaluative and iterative strategies (52% vs. 23%).

**Table 4:** Characteristics of implementation domains for all included grants, and by whether or not they were engaged research

	Number (% of total grants *N* = 103)	Number (% of grants with engagement *n* = 90)	Number (% of grants without engagement *n* = 13)
Study type			
Dissemination	0 (0)	0 (0)	0 (0)
Implementation	76 (74)	65 (72)	11 (85)
De-implementation	2 (2)	2 (2)	0 (0)
Both D&I	23 (22)	23 (26)	0 (0)
Modeling only	2 (2)	0 (0)	2 (15)
Phase of implementation^a^			
Pre-implementation	41 (40)	37 (41)	4 (31)
Describe implementation process/factors	68 (66)	60 (67)	8 (62)
Tests implementation strategy(ies)	59 (57)	55 (61)	4 (31)
Scale up	19 (18)	18 (20)	1 (8)
Sustainability	18 (17)	17 (19)	1 (8)
Measurement development	6 (6)	6 (7)	0 (0)
Mechanisms and pathways	18 (17)	17 (19)	1 (8)
Study design^a^			
Case study	3 (3)	3 (3)	0 (0)
Experimental	64 (62)	57 (63)	7 (54)
Quasi-experimental	7 (7)	6 (7)	1 (8)
Observational	36 (35)	33 (37)	3 (23)
Pre-post	6 (6)	5 (6)	1 (8)
Modeling	8 (8)	6 (7)	2 (15)
Other	8 (8)	8 (9)	0 (0)
Hybrid study			
Type 1	23 (22)	22 (24)	1 (8)
Type 2	24 (23)	18 (20)	6 (46)
Type 3	13 (13)	12 (13)	1 (8)
Not hybrid	43 (42)	38 (42)	5 (38)
Theory^a^			
Consolidated Framework for Implementation Research (CFIR)	43 (42)	38 (42)	4 (31)
Reach, Effectiveness-Adoption Implementation, Maintenance (RE-AIM) or Practical Robust Implementation and Sustainability Model (PRISM)	35 (34)	29 (32)	6 (46)
Exploration, Preparation, Implementation, Sustainment (EPIS)	12 (12)	8 (9)	2 (15)
Others	57 (55)	54 (60)	3 (23)
None	8 (8)	6 (7)	2 (15)
Implementation dtrategies^a^			
Adapt and tailor to context	50 (49)	45 (50)	5 (38)
Change infrastructure	15 (15)	13 (14)	2 (15)
Develop partner interrelationships	35 (34)	32 (36)	3 (23)
Engage consumers	37 (36)	36 (40)	1 (8)
Provide interactive assistance	32 (31)	29 (32)	3 (23)
Support clinicians/service providers	27 (26)	26 (29)	1 (8)
Train and educate partners	46 (45)	43 (48)	3 (23)
Use evaluative and iterative strategies	51 (50)	47 (52)	3 (23)
Utilize financial strategies	10 (10)	9 (10)	1 (8)
Other	6 (6)	5 (6)	1 (8)
None	6 (6)	3 (3)	3 (23)

^a^Responses not mutually exclusive

**Fig. 1:**
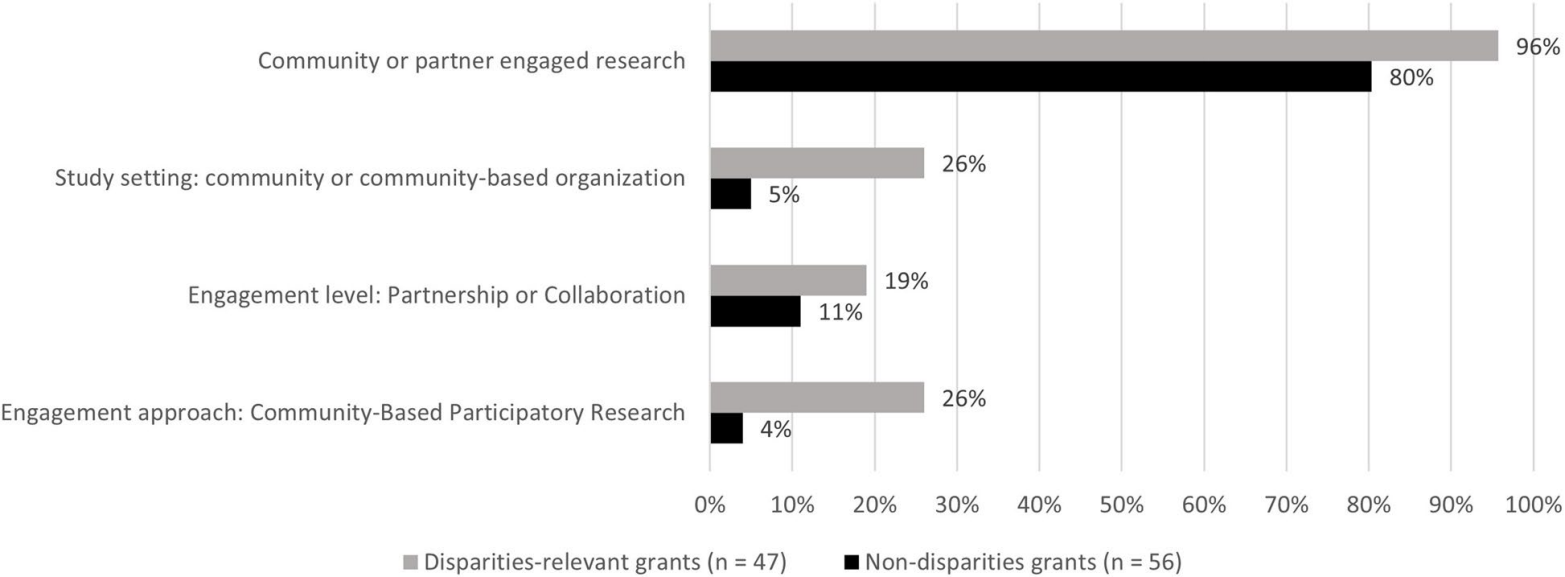
Comparison of how NIH-funded D&I research grants involve community or partner engagement by disparities relevance

In looking at themes that emerged with respect to engagement in the subset of 47 grants focused on populations with health disparities, all but two had some level of engagement (96%, *n* = 45). Compared to non-disparities studies, disparities-relevant studies were almost twice as likely to engage partners at the level of partnership or collaboration (19% vs. 11%) (Fig. 1). About 26% of disparities-focused studies were conducted in the community or at a community-based organization compared to just 5% of non-disparities studies. CBPR was the most common engagement approach described in studies with populations experiencing disparities at 26% compared with only 4% in studies not focused on disparities.

## Discussion

**Fig. 2:**
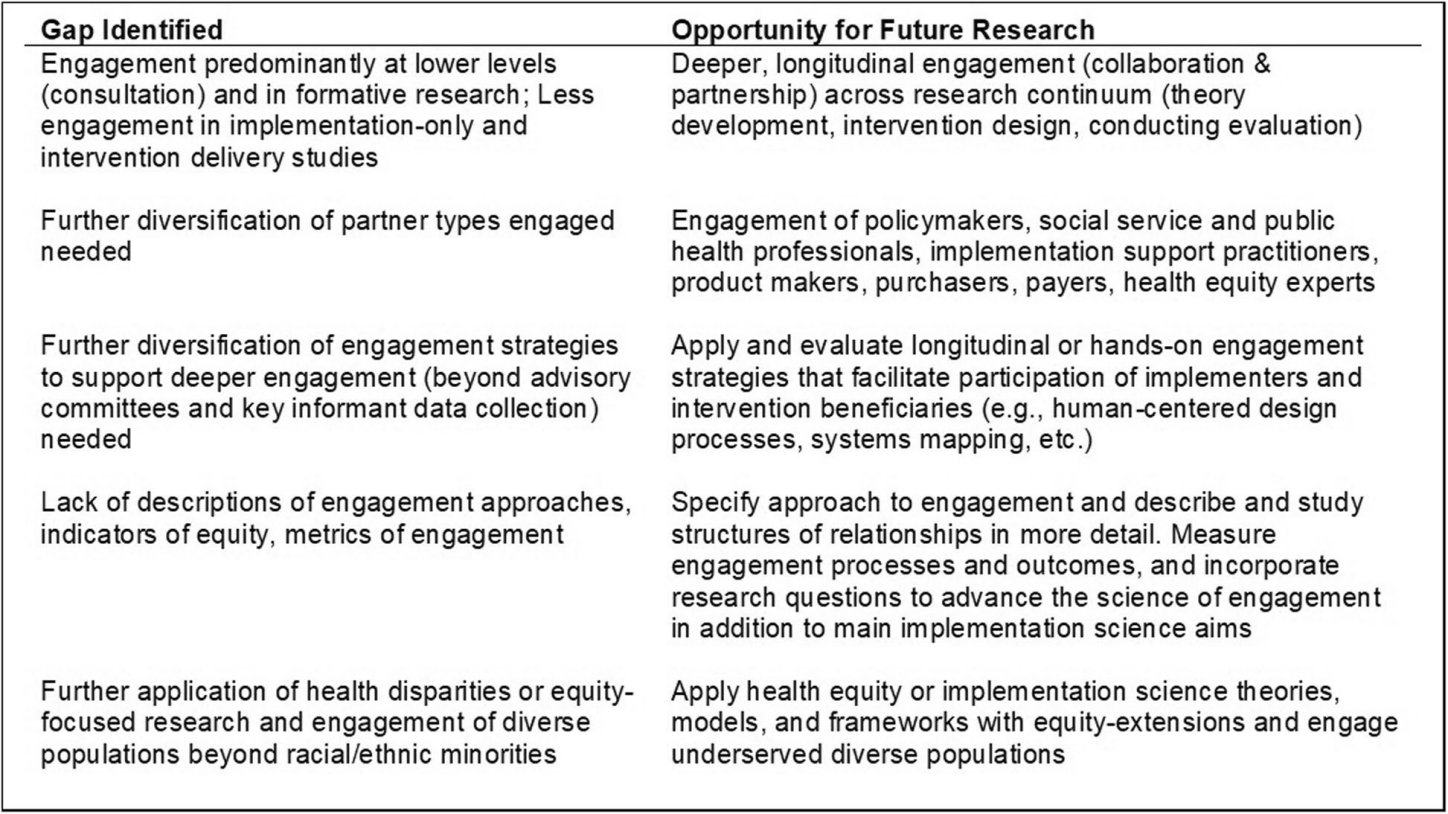
Summary of gaps and opportunities in community engaged implementation science

This analysis illuminated gaps in the application of engagement in NIH-funded D&I research and opportunities to study engagement processes and outcomes in the context of this research (Fig. 2).

For our primary research question of to what extent and how are community members and partners engaged in recent NIH-funded D&I research, it was not surprising to see that nearly nine in ten grants reviewed involved engagement in some way, given the increased attention to engaged research in recent years. However, engagement scientists might argue that the more than half of studies involving partners at the level of consultation are not truly meeting the spirit of engagement when partners are simply serving as research participants, key informants, or advisory board members. Level of engagement did not appear to vary by type of partner, though that was difficult to examine since multiple partners were often engaged in each grant. Full partnership may not be warranted or necessary in every research study, but the rigor and relevance of D&I research would likely benefit if investigators were engaging communities and partners at the higher levels of collaboration and partnership more often. This opportunity goes beyond engagement just in formative research and implementation strategy design. To fully appreciate the potential benefits of such engagement, opportunities across the research continuum will likely be necessary. For example, community and partner engagement in theory development could result in theories, models, and frameworks that are more relevant and applicable to the populations of interest. Likewise, engagement in intervention design and evaluation may lead to more effective and sustainable interventions and assessment of outcomes that matter most to communities and partners.

It was encouraging to see that multiple diverse groups of partners, on average, were engaged in each study. For intervention implementers, we found opportunities remain to increase engagement of policymakers, social service workers, public health professionals, and implementation support practitioners (e.g., knowledge brokers, technical assistance providers, practice facilitators). Further, engagement with product makers, purchasers or payers, and health equity experts could be considered to potentially increase outcomes related to “designing for dissemination” including acceptability, cost-effectiveness, scalability, and sustainability [57, 58]. Our findings were largely consistent with the findings from the review of 103 child mental health implementation studies by Triplett et al. [17]. Providers were the most commonly engaged and both analyses highlighted gaps in terms of limited depth of engagement (skewed toward consultation) and opportunities to increase engagement with policymakers, private payers, and patients/clients.

Whereas an engagement approach was not specified in one in three grants that had engagement activities, it was encouraging to see that nearly one quarter of the 90 grants with engagement were employing a practice-based research approach (23%, *n* = 22), and more than a quarter were conducting CBPR or following a generic community-engaged research approach (27%, *n* = 24). Grant applications that did not identify a specific approach were most commonly operating at the consultation level, reinforcing critiques that they are likely not meeting the true spirit of engagement. Opportunities remain for incorporating strategies that involve deeper engagement approaches with both end-user implementers and beneficiaries of innovations such as human or user centered design [59], process mapping [60], systems mapping [61], intervention mapping [62], or implementation mapping [63], and other longitudinal and hands-on strategies. Future research can explore the impact of these increasing levels of engagement and various strategies on relevant implementation outcomes.

Indicators of equitable engagement were challenging to extract and infer from the proposals, despite having access to the full narrative and related budget documents. Where indicators were apparent, partners’ influence on the research and mutual benefit of engagement were most commonly evident. Little to no description of issues of ownership, responsibilities, or power and control over the data, findings, or otherwise was found. It is possible that issues of equitable engagement emerged and were addressed over the course of the project but were not included in the proposal for several reasons: not part of grant evaluation criteria; lack of space; not yet defined with partners; thought to be irrelevant to the science. Regardless, the issues around the influence of power, responsibility, ownership, and control in engaged research and as they relate to dismantling structural drivers of social determinants of health (SDOH) are ripe for future research. Exploring power dynamics in engaged research and proactively co-creating acceptable engagement structures could enhance community participatory research [64]. In 2021, Shelton et al. [65] outlined recommendations to the D&I research field for addressing structural racism that included leveraging engagement as an essential method, assessing and addressing power differentials, and applying multi-level approaches for implementing interventions, policies, and strategies to address structural discrimination and advance health equity. In 2022, Stanton et al. [66] proposed a typology of 3 types of power that appear in implementation efforts. Discursive, epistemic, and material power respectively relate to how narratives about health and its determinants are formed, what forms of evidence are valued, and how resources are distributed. Stanton et al. [66] outlined a number of research avenues to investigate how power operates and influences implementation and health equity-related outcomes. Consistent with these recommendations, the findings of the present analysis suggest that opportunities remain to explicitly address equitable engagement in D&I research as it could influence how these types of power are established and leveraged by ensuring that a diversity of voices and experiences are included and elevated.

D&I research offers promise for more systematically applying engagement and in so doing, providing a venue for advancing the science of engagement. Distinct from participatory research that uses engagement techniques, engagement science examines the methods and outcomes of engagement in order to develop an evidence base for why engagement matters and how to do it well [67–69]. Triplett et al.’s review found that implementation outcomes most likely to be related to engagement were least reported and few of the more than 100 projects reviewed reported the impacts of engagement [17]. Certainly, studying engagement and relating it to D&I strategies or outcomes is not possible without measuring or documenting engagement in some way. Given that most grants reviewed here did not formally assess engagement, there remains a significant opportunity for D&I scientists to fully embrace engagement approaches and rigorously assess and measure these processes to ideally make an impact on the sciences of D&I and of engagement moving forward.

Our second research question strived to uncover any trends in engagement variables based on key study characteristics. There were noted missed opportunities for engaging key community members and partners in a portion of studies focused solely on implementation. Furthermore, there was a notable lack of engagement described in studies that involved delivery of an intervention; future studies could likely benefit from more active attempts to engage the implementers, decision makers, and intervention beneficiaries more substantially and to examine the impact that may have on study design, execution, and outcomes. Although there were fewer grants in total that were both D&I (*n* = 23), they all involved engagement of some sort, possibly due to the increased challenge of studying and successfully accomplishing both dissemination and implementation within a single time-limited grant. Similarly, grants involving testing strategies, scaling up, or studying sustainability were more likely to involve community or partner engagement, possibly due to the active and complex nature of these phases of implementation. In their review, Triplett et al. also found deeper engagement with more advanced phases of implementation [17]. Many implementation efforts require engagement with a decision maker to facilitate the test of change, and studying scale up and sustainability are intuitively enhanced with the involvement of partners responsible for expanding and maintaining program implementation. Similarly, case study and observational study designs appeared to be more common in engaged grants, likely because they required access through partners to collect data. In contrast, grants without engagement were more likely to utilize modeling approaches, which may not obviously require input from partners. Yet, partner engagement in modeling approaches to interventions remains a relatively unexplored area of inquiry that has the potential to make such efforts more responsive and relevant to the communities they aim to serve. Indeed, an initial effort to develop a “participatory systems dynamics modeling” approach has shown promise [70]. Logically, the implementation strategies employed in engaged grants tended to be strategies that stemmed from that engagement (e.g., engaging consumers, developing interrelationships, training). Similarly, community-situated studies all involved engagement and there was greater indication of decision making and power and control in studies at higher levels of engagement.

It was encouraging to find that nearly half of grants overall were relevant to disparities in some way. Given the potential of D&I research and engagement approaches to address SDOH and advance health equity among underserved populations who experience disparities [24, 71, 72], opportunities remain for growing the amount of disparities-related and equity-focused engaged D&I studies. In particular, there was limited engaged research with some populations experiencing health disparities such as underserved rural populations, sexual and gender minorities, and others (e.g., disability communities). With respect to engagement themes among disparities-focused grants, nearly all these grants involved engagement at some level, and they engaged partners more deeply at the levels of collaboration or partnership as compared to non-disparities focused grants. This may be in part due to the longitudinal commitment necessitated by equity-focused work as well as the noted increasing federal support for engaged research approaches to advance health equity. The disparities-focused grants were more likely to be conducted in a community setting or at a community-based organization and were more likely to involve multi-level interventions as compared to non-disparities grants. These findings perhaps indicate that D&I researchers are beginning to answer the calls to truly partner with communities and devise multi-pronged strategies that address complex problems, build capacity among D&I researchers, and incorporate equity across implementation focus areas [65, 73].

NIH has been a leader in building the field of D&I research, co-founding the Annual Conference on the Science of Dissemination and Implementation in 2007 and growing a dedicated D&I grants portfolio over nearly 20 years that is now supported by most of the Institutes, Centers, and Offices. Our analysis was limited to grants funded through the three NIH-wide D&I funding opportunities and/or reviewed by the SIHH/DIRH study section. These are investigator-initiated research awards, not necessarily related to a targeted funding opportunity requiring engagement, so this sample of grants should be representative of the range of current investigator-initiated research in the D&I field. With more than approximately $1 billion in D&I funding in fiscal year 2022 according to NIH’s categorical spending report [74], we are confident that NIH is one of the largest funders of D&I research and therefore the gaps and opportunities identified in this portfolio (Fig. 2) may extend to the broader D&I field. Consistent with gaps previously noted in the literature [17, 22, 24], we identified engagement in most grants analyzed but engagement was often not deep or rigorous, lacked a diversity of partners, and failed to evaluate engagement processes or outcomes. NIH and other funders of D&I research may wish to conduct internal analyses of initiatives that have promoted community and partner engagement generally, and specifically in D&I research, to identify successful strategies to use in future funding opportunities. In addition, other funding organizations, such as PCORI, may offer lessons learned about funding opportunity language, review criteria, investigator training, and other practices that address noted gaps in the application of engagement science within D&I research.

### Strengths and limitations

This portfolio analysis had several strengths, including the large sample of grants, the use of dual coders, and a structured codebook. In addition, as an internal NIH project, coders had access to the full grant proposal narrative including the research summary and budget documents to be able to code more accurately.

Yet, important limitations were present as well. The inclusion criteria were limited to a specific set of funding opportunities and a single study section; this likely omitted some D&I research funded across NIH through other mechanisms and study sections. However, the included grants do incorporate the single largest portion of individual investigator-initiated D&I research grants supported by the NIH. Another limitation is that NIH is not the only funder of D&I research, although possibly the primary one, and because this analysis was limited to a selection of NIH-funded grants, we cannot speak to the work funded by others. Given the literature cited in the introduction and discussion has similar themes to our findings, it is likely that this portfolio analysis is fairly representative to the state of the field at large.

Another limitation is that coders reviewed grant proposals, which may or may not be a true reflection of study activities implemented once funded. Some engagement variables coded, such as approach and equity indicators, are elements that are not required to be specified or described in proposals, potentially leading to an overcount of “not specified/described.” Finally, despite training coders, developing a thorough codebook with definitions, and providing support throughout the project, coding is subject to human judgement and manual data entry errors, though this was mitigated by dual coding to agreement.

## Conclusions

Based on this portfolio analysis, the D&I research field appears to regularly integrate community and partner engagement approaches and strategies, though opportunities remain to deepen engagement and diversify partners. Further, to meaningfully advance health equity, there are critical opportunities for D&I research that leverages community and partner engagement to address health disparities, especially among underserved populations. Through this increased attention to engagement within D&I research, new opportunities to study engagement-related research questions may be pursued that will likely increase the rigor, relevance, and impact of both engagement science and D&I research.

## Data Availability

The datasets generated and analyzed during the current study are not publicly available due to the confidential nature of grant proposals but a limited dataset is available from the corresponding author on reasonable request.

## Abbreviations

CBPR: Community-Based Participatory Research
COVID-19: Coronavirus-2019
D&I: Dissemination and implementation
DIRH: Dissemination and Implementation Research in Health
NIH: National Institutes of Health
SDOH: Social determinants of health
SIHH: Science of Implementation in Health and Healthcare
TIDIRC: Training in Dissemination and Implementation Research in Cancer
